# On the History of Hepatic Vein Catheterization

**DOI:** 10.1111/liv.70827

**Published:** 2026-08-14

**Authors:** Jens Henrik Henriksen, Flemming Bendtsen, Søren Møller

**Affiliations:** ^1^ Clinical Physiology 260, Center of Functional and Diagnostic Imaging and Research, Faculty of Health Sciences Hvidovre Hospital, University of Copenhagen Copenhagen Denmark; ^2^ Gastrounit, Medical Section 360 Amager‐Hvidovre University Hospital Copenhagen Denmark; ^3^ Department of Clinical Medicine University of Copenhagen Copenhagen Denmark

**Keywords:** cirrhosis, hepatic blood flow, indirect Fick‐method, portal hypertension, TIPS, transvenous liver biopsy

## Abstract

Hepatic vein catheterization (HVC) in man was first performed 80 years ago in the wake of right‐sided heart catheterization. Several new methods, recognitions and concepts followed, especially the indirect Fick‐method for determination of splanchnic blood flow. Five years passed before pressure measurements were performed and routine HVCs were done in liver patients. Later came splanchnic kinetics of test substances, dyes, metabolites, hormones and bioactive material and counter current wedged and balloon occlusion pressure. HVC has been a prerequisite for our understanding of the pathogenesis of portal hypertension and variceal bleeding leading to serial measurements, stratification, quantification and treatment of portal hypertension, and transvenous liver biopsy and portosystemic stent insertion (TIPS) followed. Measurements and pathophysiology of formation and amelioration of ascites advanced with HVC, likewise pathophysiology of known and new diseases in other organs, secondary to liver disease (kidney, heart, lung, systemic circulation). The hepatic venous pressure gradient (HVPG, i.e., wedged‐to‐free hepatic venous pressure) is today considered the gold standard for portal pressure measurement in intrasinusoidal and postsinusoidal portal hypertension. In spite of substantial developments in route, catheter type, pressure measurements and ‐registration, fluoroscopy, transvascular technique, etc. the basic concepts are the same today. New advanced technology, materials, imaging techniques and IT‐power have together with the inborn dynamics of the catheterization given a progressive development, and there are potentials of further refinement and applications in science, clinical physiology, gastroenterology and hepatology.

## Key Points

- Hepatic vein catheterization (HVC) was first performed in man 80 years ago in the wake of right‐sided heart catheterization.
- HVC allows routine measurements of hepatic pressures, blood flows, and to obtain elective blood samples of bioactive material, test substances, metabolites, etc.
- Pathophysiology of portal hypertension, ascites formation and secondary organ disease, like cirrhotic cardiomyopathy, has been investigated with HVC.
- Transjugular intrahepatic portosystemic stent shunt (TIPS) and transvenous liver biopsy were developed from HVC.
- Today the hepatic venous pressure gradient (HVPG) is the gold standard in diagnostics and control of intra‐ and postsinusoidal portal hypertension.

## Lay Summary

- Catheterization of liver veins, first performed 80 years ago, allows measurement of liver blood pressure and flow, and analysis of organ blood and tissue samples.
- The catheterization technique has contributed substantially to diagnostics, understanding of disease development, and therapy of chronic liver failure and elevated pressure in gastro‐intestinal veins. The procedure is today the gold standard for pressure measurements in the liver.

**Abbreviations**
ANP: atrial natriuretic peptide
BNP: brain natriuretic peptide
BSP: bromsulphalein
CNP: C‐type natriuretic peptide
eNOS: endothelial nitric oxide synthase
ET‐1: endothelin‐1
ET‐3: endothelin‐3
FHVP: free hepatic venous pressure
HVC: hepatic vein catheterization
HVPG: hepatic venous pressure gradient
ICG: indocyanine green
NA: noradrenaline
proBNP: propeptide of BNP
RAAS: renin angiotensin aldosterone system
TIPS: transjugular intrahepatic portosystemic stent
TJALB: transjugulo atrial liver biopsy
VIP: vasoactive intestinal peptide
WHVP: wedged hepatic venous pressure

## 1 Introduction

Hepatic vein catheterization (HVC) is, together with liver biopsy, sonographic examination, other imaging modalities, like CT‐, PET‐ and MR‐scanning, endoscopy, transient elastography and immunological techniques, among the most important progresses in hepatology in relation to diagnosis, treatment and pathophysiological mechanisms behind portal hypertension and its complications [1]. The catheterization technique has played a major role in experimental and clinical decision making, but in the present context, it has especially revolutionized the diagnostics of portal hypertension [2]. The HVC has undergone significant progress, although uneven in the passage of time [3].

This survey will focus on the historical development with emphasis on pioneers, methods and techniques, and results which have had significant value for the understanding of the pathophysiology of portal hypertension, hemodynamic consequences and complications for the patients and for the medical specialties hepatology and gastroenterology.

### 1.1 From Right Heart Catheterization to Hepatic Vein Catheterization

The early HVC followed the wake of the right‐sided heart catheterization in the 1940s and 1950s [4]. Therefore, a history of HVC should start with a short survey of the right‐sided heart catheterization and its dramatic start.

In 1929, the young German physician Werner Forssmann (1904–1979) catheterized his own heart [5]. This was preceded by attempts to catheterize dead human bodies. Forssmann succeeded and documented the catheterization of his right heart by an X‐ray. Initially, he received little honour but a lot of criticism. This was due to major scepticism in the lay public as well as professionally, insufficient contrast media, and too long exposure time to obtain X‐ray pictures of the heart cavities [6, 7].

In the mid 1930s, André F. Cournand (1895–1988) and Dickinson W. Richards (1895–1973), two clinical pulmonary physiologists at Bellevue Hospital, Chest Service, Columbia University, New York, were interested in gas exchange and the uneven composition of the air in the lungs of patients with pulmonary diseases. In this context, the need for mixed venous blood was imminent [4]. They prepared for some time to put a catheter into the right atrium of such patients, but experiments with rebreathing brought them some way, and they hesitated. The incitement should come from another direction.

**FIGURE 1:**
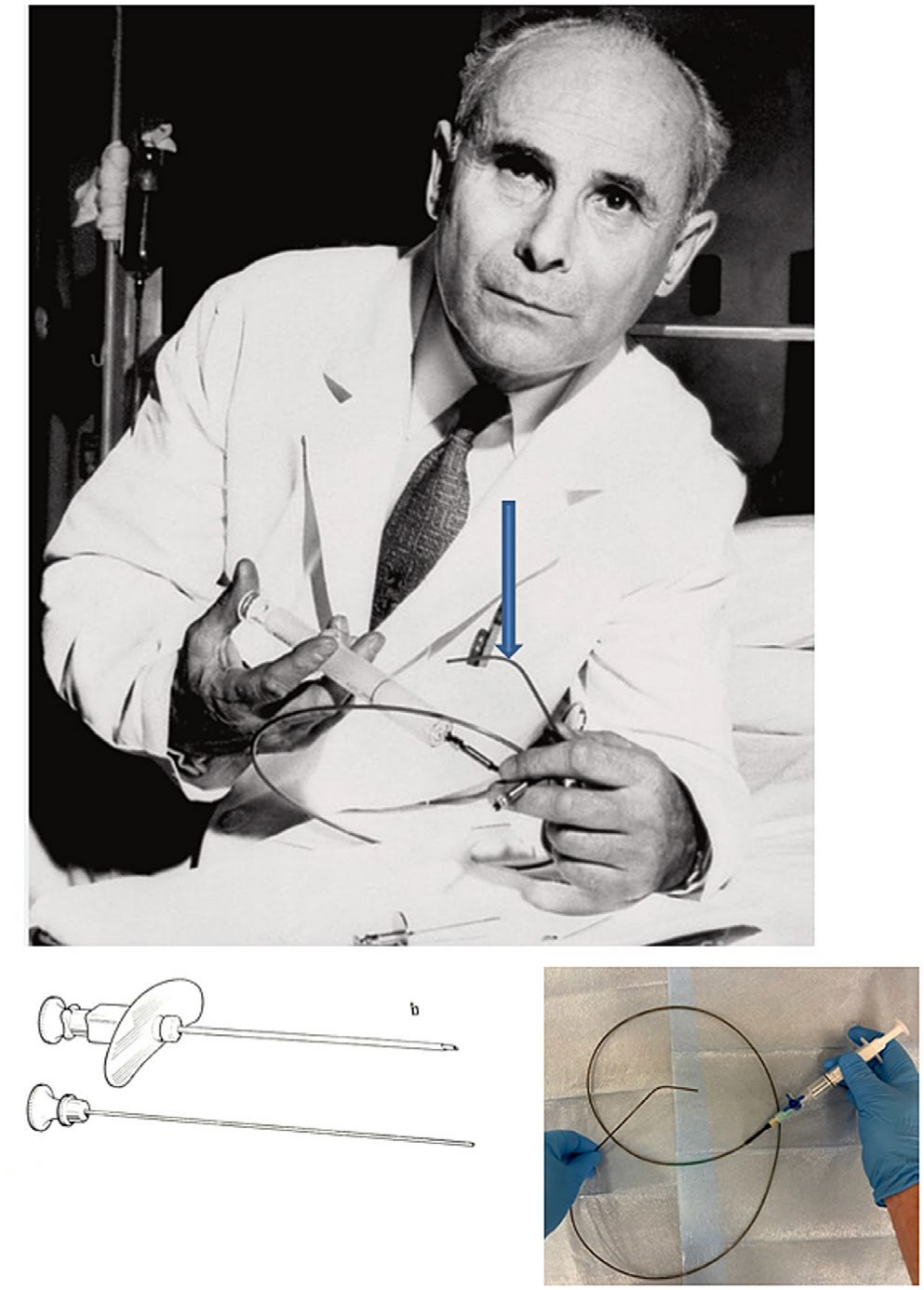
André Cournand demonstrates the Cournand catheter with a glass syringe. The arrow indicates the angled tip, a shape ideal for passing through the right heart ventricle and for catheterization of right hepatic veins with wedging. In the bottom Cournand's flanged arterial needle with obturator (left) and a modern edition of the Cournand catheter (right). From authors' archive.

Homer Smith (1895–1962), professor of kidney physiology at New York University, wished to compare in man determination of cardiac output by ballistocardiography^1^ to that of Fick's method [4, 6, 8]. The latter implied determination of oxygen uptake in the lungs and oxygen content in mixed venous and arterial blood. This method had not been performed in man before, but was standard in animal experiments. It has been told that Cournand and Richards were forced by Smith and the young physiologist Stanley E. Bradley (1913–1999) to place a catheter in the human heart in this comparative investigation [4, 6]. The great value of the work [9], published in 1941, turned out to be the establishment of heart catheterization as a reliable and safe procedure with results that could be reproduced in the determination of cardiac output and analysis of regional blood composition [10, 11]. The first type of catheter to be inserted into the human heart was a ureter catheter. Later on Cournand developed the so‐called ‘Cournand catheter’ (Figure 1) with the optimal curvature for passage through the right heart chamber out in the pulmonary artery [4], and it turned out also to catheterize the hepatic veins quite easily. Bradley became a coinvestigator and author on these studies^2^ [12, 13, 14].

In the early 1940s, heart catheterization was solely performed in New York. In 1943, the technique was brought to France by Jean Lenègre (1904–1972) and Pierre Maurice (1916–2005) [15] and to England by the heart physician (later Sir) John McMichael (1904–1973) and the Scottish‐English physiologist Edward P. Sharpy‐Schäfer (1908–1963) [16]. Both groups met clinical scepticism and resistance. By the middle of the 1940s heart catheterization was still experimental and investigative for a medical elite at university departments. However, the technique was from then on quickly accepted and spread to Scandinavia and other areas in the USA [4, 17, 18].

## 2 Hepatic Vein Catheterization (HVC)

Although Stanley E. Bradley is considered ‘the father of hepatic vein catheterization’, he was neither the first to draw a blood sample from a hepatic vein nor the first to measure the pressure here. Bradley belonged to the group who started catheterization in man [4, 6, 12, 13], he brought it to other centres in the USA [4], and he established the *indirect Fick‐method* to measure splanchnic/hepatic blood flow [19], analysed the method in detail, and determined several physiological and pathophysiological variables in man and animals [20, 21, 22, 23]. He analysed processes in the liver parenchyma and biliary excretion [24, 25] and described fundamental kinetics of splanchnic/hepatic mean circulatory times [26]. In addition he trained several young doctors and researchers [27, 28], as described later.

**FIGURE 2:**
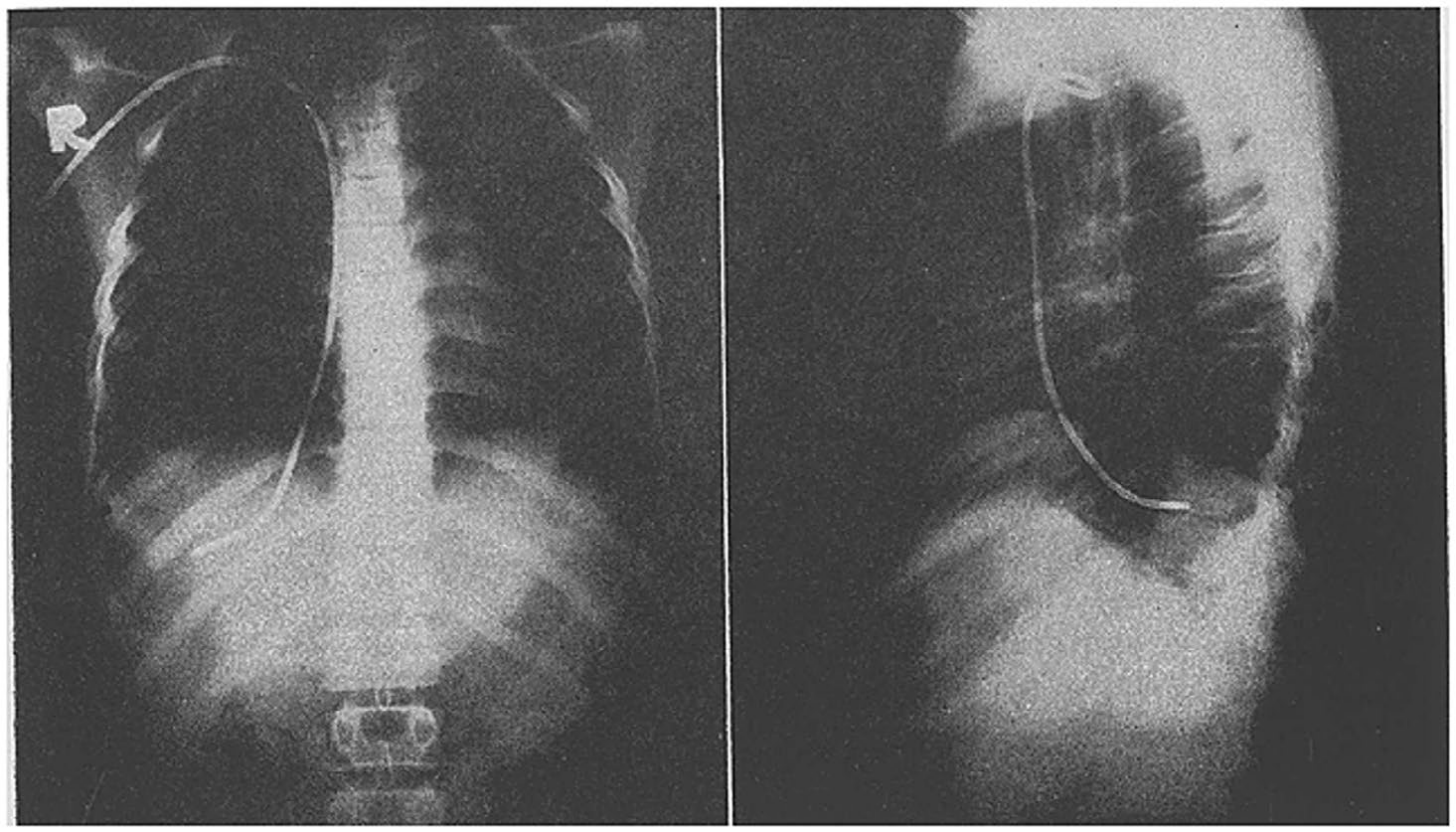
The first published X‐ray of hepatic vein catheterization via right antecubital vein, through the right atrium to a right hepatic vein. The catheter is a ureter‐catheter with a 30° Cournand‐angled tip. Left: anterior–posterior, right: site‐projection. Warren and Bannon 1944 [29]. From authors' archive.

**FIGURE 3:**
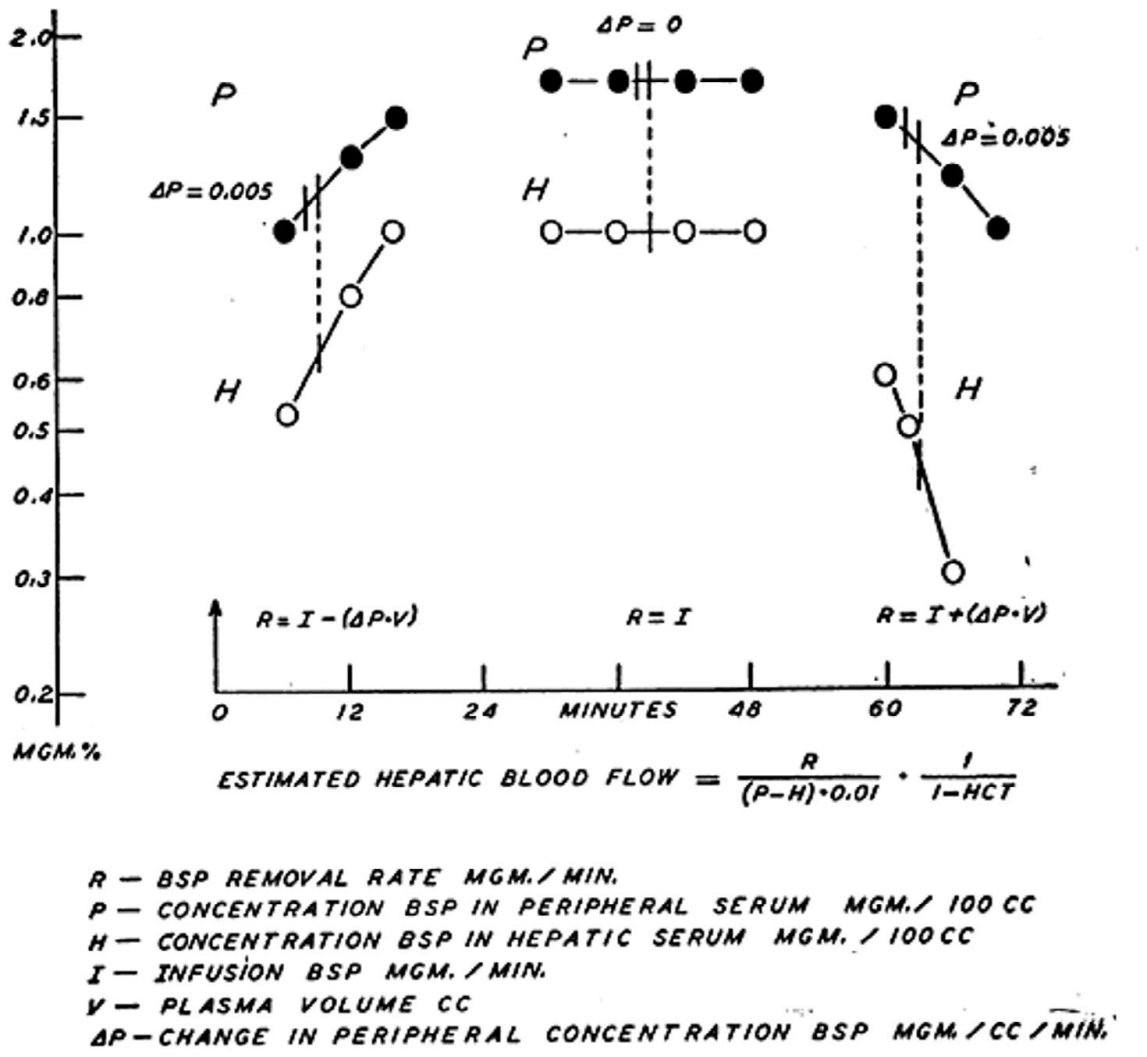
Stanley Bradley's illustration of the *indirect Fick‐principle* for measurement of hepatic blood flow with bromsulphalein (BSP) infusion. The left and right parts of the figure illustrate unsteady state with increasing and decreasing, respectively, circulating concentration of BSP and correction of infusion rate. Redrawn from Bradley 1945 [19].

The first report on a catheter in a hepatic vein was by Dr. James V. Warren (1915–1990) at Grady Memorial Hospital and Emory University School of Medicine, Atlanta, Georgia. Warren and coworker Emmett S. Bannon published a short notice on obtaining blood samples from hepatic veins in *Proceedings of the Society of Experimental Biology and Medicine* in 1944 [29]. They described in detail the procedure of hepatic vein catheterization with a 8–9 French ureter catheters with 30° Cournand angled tip, based on their experience of 75 patients, see Figure 2. They also gave a gratitude to André Cournand for their fundamental training. They found that haematocrit and haemoglobin were similar in hepatic vein and right atrium, but oxygen content, bacterial colony counts and glucose differed. The following year, Bradley and co‐workers published their large and very impressive paper on the measurement of hepatic blood flow in man [19]. This was a remarkable investigation with the well described technique of HVC with a Cournand catheter through the right atrium in 23 persons with normal hepatic function. He also settled the principle of *indirect Fick‐method*, see Figure 3. The use of dyes (like bromsulphalein), which was known from animal experiments to bind to plasma proteins, and to be exclusively eliminated by the liver into the bile, was established. The normal liver blood flow was mean 1.497 L per minute with a range of 1.085–1.845 L per minute. The representativeness of hepatic blood sampling was evaluated by obtaining blood samples from veins of different locations in the right liver lobe. The publication by Bradley in 1945 was the first of several [20, 21, 22, 23, 24, 25, 26]. However, neither Warren's nor Bradley's publications in the mid 1940s mentioned the possibility of pressure measurements in hepatic veins [19, 29].

### 2.1 Early Pressure Measurements

In 1947, Cournand and co‐workers had measured the blood pressure in the left atrium and the pulmonary veins in a patient with an atrial septal defect [30]. Two years later, Hellems, Haynes and Dexter measured what was called *pulmonary capillary pressure* in man [31]. By wedging the catheter tip into a small pulmonary artery, the circulation in that part of the lung was stopped and the vessel was so to speak applied as an elongating of the catheter tip, with the result that the pressure was determined in the pulmonary capillary network and venules, a pressure that turned out to be almost identical to the pressure in the pulmonary veins and left atrium. The pressures were measured by a Hamilton manometer [14]. The results were confirmed by Hans Lagerlöf and Lars Werkö in Sweden also in 1949 [17]. Although the wedging in the lung circulation was performed downstream in the direction of pulmonary blood flow, this method turned out to be a general technique for measuring pressures in capillaries and beyond the capillary network.

### 2.2 Pressure Measurements During Hepatic Vein Catheterization

The level of the portal pressure in animals and normal subjects was well known from numerous direct measurements during surgical procedures under laparotomy. This also applied to patients with liver diseases, where direct measurements during open abdominal surgery had revealed pressures from 10 to over 40 mmHg in patients with portal hypertension. However, a method for minimal invasive determination of portal pressure was still missing.

The technique of ‘catheter wedging’ from the lung measurements was quickly applied in 1950 in the liver of dogs by E.W. Friedman and R.S. Weiner, who found almost similar pressures by catheter wedging in the liver and by direct measurements in the portal vein [32]. Moreover, when manipulating the vascular pressures by volume change and adrenaline injections, the pressures changed concordantly at the two measuring locations. Similar results were obtained by W. Jape Taylor (1924–2011) and coworker J.D. Myers from Duke University, Durham, North Carolina in cats and patients in 1951 [33, 34]. The following year, H.R. Bierman and co‐workers reported results of pressure measurements from a direct percutaneous transhepatic puncture of the portal vein [35].

**FIGURE 4:**
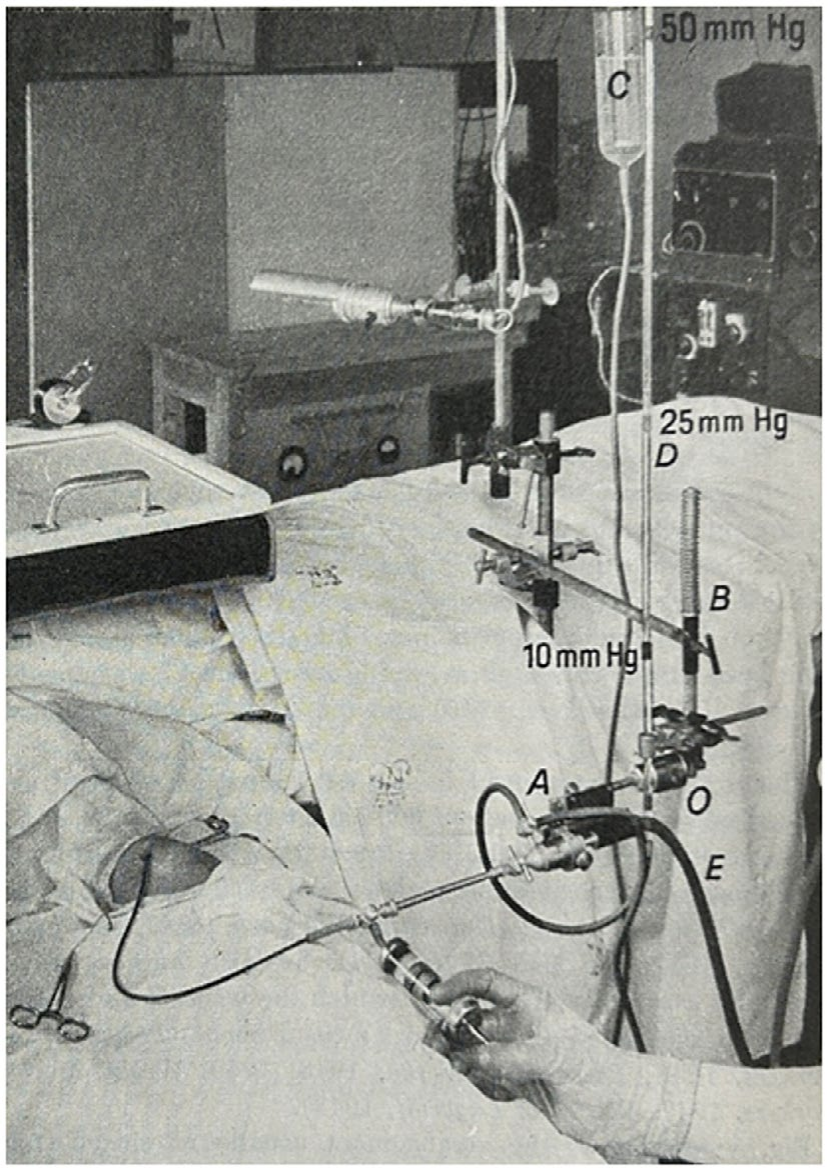
Hepatic vein catheterization set up in early 1950s. The Cournand catheter is passed via a right antecubital vein. (A) Tybjærg Hansen‐condensor manometer, (B) scaffold, (C) line for irrigation, (D) stand for calibration bottles and (E) manometer cable. The characteristic fluoroscopic box is in front of the patient to the left. From authors' archive.

Telfer B. Reynolds (1921–2004) from University of Southern California was in the early 1950s a research fellow at the Royal Free Hospital, London with Professor (later Dame) Sheila Sherlock (1918–2001). Here, HVC was already a routine procedure for the determination of splanchnic blood flow and metabolic studies [36, 37]. Paton, Reynolds and Sherlock reported their results on pressure measurements during HVC in patients with portal hypertension in the Lancet in 1953 [38], and Hans Krook from Lund, Sweden published similar findings, also in 1953 [39], followed by Hansen, Tygstrup and Winkler from Copenhagen in 1954 [40], see catheterization set up on Figure 4.

Several groups were now performing the wedging procedure during HVC [41, 42, 43, 44, 45]. The results of wedged hepatic vein pressure measurement on the one hand were compared with simultaneous measurements of splenic pulp pressure after percutaneous needle puncture, simultaneous and serial measurements of portal pressure during laparotomy on the other hand in patients without liver disease, patients with cirrhosis (+/− ascites, bleeding, jaundice), patients with non‐cirrhotic and extrahepatic portal hypertension, and in patients with congestive heart failure. The main results of these studies were as follows: (1) The original term ‘hepatic sinusoidal pressure’ should be substituted by ‘wedged hepatic vein pressure’ (WHVP); (2) WHVP was close to be identical with the directly measured portal venous pressures (although some results showed WHVP to be slightly lower, other to be slightly higher); (3) higher values of WHVP were found in patients with bleeding from oesophageal varices and severe jaundice, but unclear results were found in patients with ascites; (4) WHVP was normal in non‐cirrhotic and extrahepatic portal hypertension; (5) Patients with congestive heart failure had elevated WHVP to the same degree as the elevated right atrial pressure, but with normal pressure gradient; (6) patients with end‐to‐side portocaval anastomosis had elevated WHVP, but a lower or normal portal pressure; and (7) an increased pressure gradient between the inferior vena cava and the right atrium was present in patients with cirrhosis, especially those with ascites.^3^ Some serial measurements of WHVP showed reduction of this pressure with clinical improvement. Discussions of pressure reference point (5 cm below sternum, mid‐axillary line, 5 cm above the column, the right atrium, the inferior vena cava) were frequent, but establishing of the wedged‐to‐free hepatic vein pressure or hepatic venous pressure gradient (HVPG) as a standard was yet to come (see later).

### 2.3 Back to Bradley: Hepatic Blood Flow and Splanchnic Volume Determined During HVC

Bradley moved in 1947 to Boston, where he introduced the heart catheterization technique and also performed hepatic and renal vein catheterizations [4]. In 1948, he returned to Columbia. Bradley and co‐workers continued their studies on the determination of hepatic blood flow in animals and man [20, 21, 22, 23]. Several issues were investigated: Hepatic extraction of dyes, splanchnic oxygen consumption, oxygen extraction, the effect of several manoeuvers, like upright position, volume reduction and volume expansion, and exercise were investigated. Patients with different types of liver diseases were also studied [23, 25].

The most exceptional result was a kinetic investigation of transit times and splanchnic volumes [26]. Bradley created a new and highly original type of kinetics by intravenous injection of radioiodine labelled human serum albumin and subsequent quick sampling from aorta/femoral artery and hepatic vein. By determination of the time course of the tracer inlet into the splanchnic system (artery) and the time course of the tracer outlet (hepatic vein) in addition to an equilibrium measurement, it was possible to derive the mean circulation time of the substance and thereby, of that of plasma and red blood cells.^4^ A separate measurement of splanchnic/hepatic blood flow, together with the mean circulation time, would lead to the splanchnic blood volume (volume = mean circulation time × flow) [47]. Later, other investigators have applied Bradley's principle to determine the splanchnic haematocrit. There is ‘extra’ plasma in the splanchnic/hepatic system, which may be located in the space of Disse and in some parts of the portal system with laminar streams in the small blood vessels.^5^ Sherlock and co‐workers invented an analogy to Bradley's technique by tracer injection directly into the splenic pulp during combined splenoportography and hepatic vein catheterization and subsequent estimation of the size of the collateral blood flow in patients with portal hypertension [52]. This was a highly intellectual approach, but it was not applied widely because of the limited use of splenoportography.

Bradley focused on the kinetics of bromsulphalein [24, 25]. In normal subjects and patients with liver disease, he determined the *T*_max_ and the volume of distribution inside the liver (*V*_D_). These variables were changed in characteristic patterns in patients with parenchymal dysfunction and patients with different types of cholestasis. In the mid 1950s, Bradley became leader of a large department of physiology at Columbia University. His last paper with Cournand was on splanchnic blood flow and volume during exercise in 1956 [21], the year Forssmann, Cournand and Richards received the Noble Prize in Physiology and Medicine for their discoveries concerning heart catheterization and pathophysiological changes in the circulatory system. Rudolf Preisig (1929–2017) and Roger Williams (1931–2020) were research fellows with Bradley in the 1960s [28]. Williams has described this sojourn as a very interesting and profitable time with much enthusiasm and exciting scientific results [53].

It became clear that there were some problems with the day‐to‐day variation of the biliary excretion of bromsulphalein with a possible entero‐hepatic recirculation^6^ [54]. In 1957, Fox and co‐workers had introduced a new dye for cardiac output determination: Indocyanine green (Cardio Greene), which turned out to be exclusively excreted by biliary secretion [55]. In 1960, Kjeld Winkler (1925–1998) and Niels Tygstrup (1925–2007) published their comparison of hepatic blood flow determined by bromsulphalein and indocyanine green [56]. They investigated both patients without liver disease and patients with cirrhosis. In both groups, there was a rather close agreement between the two methods. Indocyanine green had an analytic advantage (owing to spectral absorption lines in plasma) and the day‐to‐day variation in hepatic excretion turned out to be smaller than that of bromsulphalein. Winkler and Tygstrup compared indocyanine green, ethanol, galactose, and bromsulphalein for hepatic blood flow determination [57]. The comparison was in favour of indocyanine green, and this substance was studied intensively in the following years [58], and is still used today. Winkler also determined hepatic blood flow by a method independent of hepatic excretion [59]. By constant infusion of a substance into the splanchnic system (through the superior mesenteric artery), elimination by the kidneys (^131^I‐hippurane), and measurements in systemic artery and hepatic vein, it was possible by a *reversion of the indirect Fick‐method* to determine hepatic blood flow [59].

## 3 Hepatic Vein Catheterization in the 1960s and 1970s

In order to avoid the minor surgical cut in the antecubital region for introducing the catheter tip into the basilic vein, the Swedish radiologist Sven Ivar Seldinger (1921–1998), in 1953, invented a brilliant percutaneous technique for atraumatic catheterization of arteries as well as veins [60]. Later, this technique became routine all over the world. However, it remained relatively unknown until Ricketts and Abrams published their experience with the method in 1962 [61], and it took almost another 10 years before it reached the liver hemodynamic laboratories.

Most HVC were still performed with a relatively large calibre Cournand catheter (8–9 F) from an arm vein, through the right atrium and further into a right hepatic veins [62]. This approach secured a stable and relatively easy wedging. The criteria for obtaining a correct wedged hepatic vein pressure are summarized in Table 1. When an arterial line was needed (e.g., determination of hepatic blood flow, galactose elimination capacity or indocyanine green clearance), the standard procedure was still a Cournand needle (Figure 1) or one of its numerous variants. Later, small polyethylene catheters became routine.

**TABLE 1:** Criteria for correct wedging of catheter in hepatic vein during pressure measurement in the 1950s.

Withdrawal of the catheter tip from the wedged position gives a very characteristic slip of the catheter, when the tip moves away from the hepatic vein/venule. (This is only present in vivo, not in dead corps, neither animals nor man).
There is an abrupt drop in pressure from the wedged to the free catheter position.
Wedged hepatic venous pressures vary only 1–2 (3) mmHg in different wedged catheter positions.
Repeated wedging of the catheter in the same position gives the same pressure measurement.
Injection of a small amount of saline in the wedged position will increase the pressure, but it will subsequently return to the same value as before the injection.
Injection of a small amount of radiopaque medium with the catheter in correct wedged position will clear very slowly from the tissue.

*Source:* From [31, 33, 34, 37, 38, 42, 43, 44].

In the 1960s, hepatic vein catheterization and adjacent techniques were performed as routine or investigative procedures in several centres [63, 64, 65, 66]: London, Paris, Munich, Bern, Copenhagen, Lund, Stockholm, Boston, New York, Los Angeles, New Haven, etc. The procedure was often performed before, during and after surgical porto‐caval anastomosis. Moreover, the different treatments could reduce the portal pressure more or less efficiently; sometimes the reduction was spontaneously [67]. Several research projects were carried out, for example, shunts, metabolism of amino acids, glycerol and ethanol, new radioactive tracers, etc. [68, 69, 70, 71].

### 3.1 The Systemic and Splanchnic Circulation in Cirrhosis and Portal Hypertension

In 1946, Perera reported that the plasma volume was increased in patients with cirrhosis [72]. In 1953, Walter Abelman (1921–2011) from Harvard Medical School, Boston and colleague Henry Kowalski (1924–2010) found that clinical elements like warm extremities, cutaneous vascular spiders, wide pulse pressure, capillary pulsations in nailbed, etc., could suggest that patients with cirrhosis might have an increased cardiac output [73]. They investigated 22 cirrhotic patients with the colour dilution technique^7^ and found that the cardiac output was increased in one third of the patients. This also held true for heart rate and stroke volume (interestingly, they also found the QT‐interval prolonged). Later they found higher values of cardiac output in patients with ascites, but no significant effect of paracentesis [75]. During exercise, heart rate, stroke volume and cardiac output increased almost as in normal subjects. They did not report on the size of plasma volume. In 1958, Murray, Dawson and Sherlock investigated patients with cirrhosis and portal hypertension, extrahepatic portal hypertension, biliary cirrhosis and normal subjects [76]. The patients with cirrhosis had a hyperdynamic systemic circulation with increased cardiac output, plasma volume, heart rate and stroke volume, but in extrahepatic portal hypertension and biliary cirrhosis the cardiac output and plasma volume were normal. It was concluded that the peripheral vasodilatation was pronounced, but it was not owing to portal systemic shunting, because this was as prominent in the patients with extrahepatic portal hypertension with normal cardiac output. The pathophysiology behind the hyperdynamic systemic circulation remained unknown. Low arterial oxygen saturation in some patients was most likely due to arteriovenous communications in the lungs. Although these studies could be performed with a peripheral venous line and an arterial needle, several studies were performed in connection with a HVC.

In 1963, the Mount Sinai pathologists Fenton Shaffner (1920–2000) and Hans Popper (1903–1988) found in patients with cirrhosis that the sinusoidal wall in the liver was changed in the direction of more tight vessels with a partial basement membrane and a narrowing of the microvasculature, the so‐called capillarization^8^ [77]. In addition, there was an increased amount of collagen in the Space of Disse. These findings were in keeping with an increased hepatic hemodynamic resistance in cirrhotic patients with portal hypertension and a compromised exchange of various substances. Vascular changes and increased microvascular pressure might also explain why some patients developed ascites, while others with equally high portal/sinusoidal pressure did not. At that time ascites formation was considered, by the traditional or classic view, as an imbalance in the Starling forces: Increased hydrostatic and reduced colloid osmotic (oncotic) pressures. This was reviewed by Sherlock and Stanley Shaldon (1931–2013) in 1963 [78]. It was a problem that early as well as recent measurements of plasma volume in advanced cirrhosis had shown elevated values [72, 76]. However, this could at least in part be explained by anaemia and the congested splanchnic circulation, and the concept of ‘effective’ (non‐splanchnic) plasma volume was introduced. Fred L. Lieberman and Reynolds focused during HVC on the measurement of wedged hepatic vein pressure and plasma volume with respect to artefacts, but found that only a small amount of plasma tracer was lost to the ascitic fluid and lymph [51]. Thus, the increased plasma volume in cirrhosis was not erroneously overestimated. Moreover, by serial determinations of plasma volume they could not show an increase in plasma volume when ascites disappeared—which was to be expected if a reduced ‘effective’ plasma volume was restored [79]. In 1970, Lieberman, Denison and Reynolds proposed the so‐called ‘overflow theory’ of ascites formation [80], according to which renal retention of sodium and water will increase sinusoidal pressure and expand the plasma volume, and fluid overflow into the peritoneal cavity. The different theories of ascites formation were going to separate researchers in many years to come.

The application of tracer injection and hepatic vein catheter‐sampling to study hepatic circulation was first reported by Carl A. Goresky (1932–1996) from McGill University, Montreal, Canada in the mid 1960s [81]. Later, Pierre‐Michel Huet and co‐workers used this technique together with hepatic and umbilicoportal vein catherization, to measure the portal fraction of hepatic blood flow prior to surgical portocaval anastomosis [82]. By use of multiple indicators they reported progressive microvascular capillarization and large vessel shunting, along with progression of cirrhosis [83].

In 1972, Roberto J. Groszmann (1939–2021) and the cardiologist at Georgetown University, Jay L. Cohn, injected ^131^I‐albumin into the splenic artery, the superior mesenteric artery and the hepatic artery of patients with cirrhosis and collected the indicator dilution samples from a catheter in a hepatic vein [84, 85, 86]. From the indicator dilution curves,^9^ they derived the segmental blood flows, the mean circulation times, and the respective blood volumes. The porto‐systemic shunting from both the splenic and superior mesenteric inlet amounted to more than 50% of total blood flow. The mean circulation time, which may be considered as a ratio between blood volume and blood flow, was substantially reduced. This indicated that the ‘splanchnic blood pooling’ in cirrhosis was not simply a result of increased hepatic vascular resistance, but in the main caused by the elevated splanchnic inflow of blood. This was a major new insight, the substantial significance of which was not immediately realized by Groszmann and Cohn.

HVC was applied by John Wahren from the Karolinska Institute, Huddinge, Sweden for the study of splanchnic glucose and amino acid metabolism in normal man, in patients with diabetes and for organ turnover of hormones [89, 90].

## 4 Transjugular Hepatic Vein Catheterization and Liver Biopsy

**FIGURE 5:**
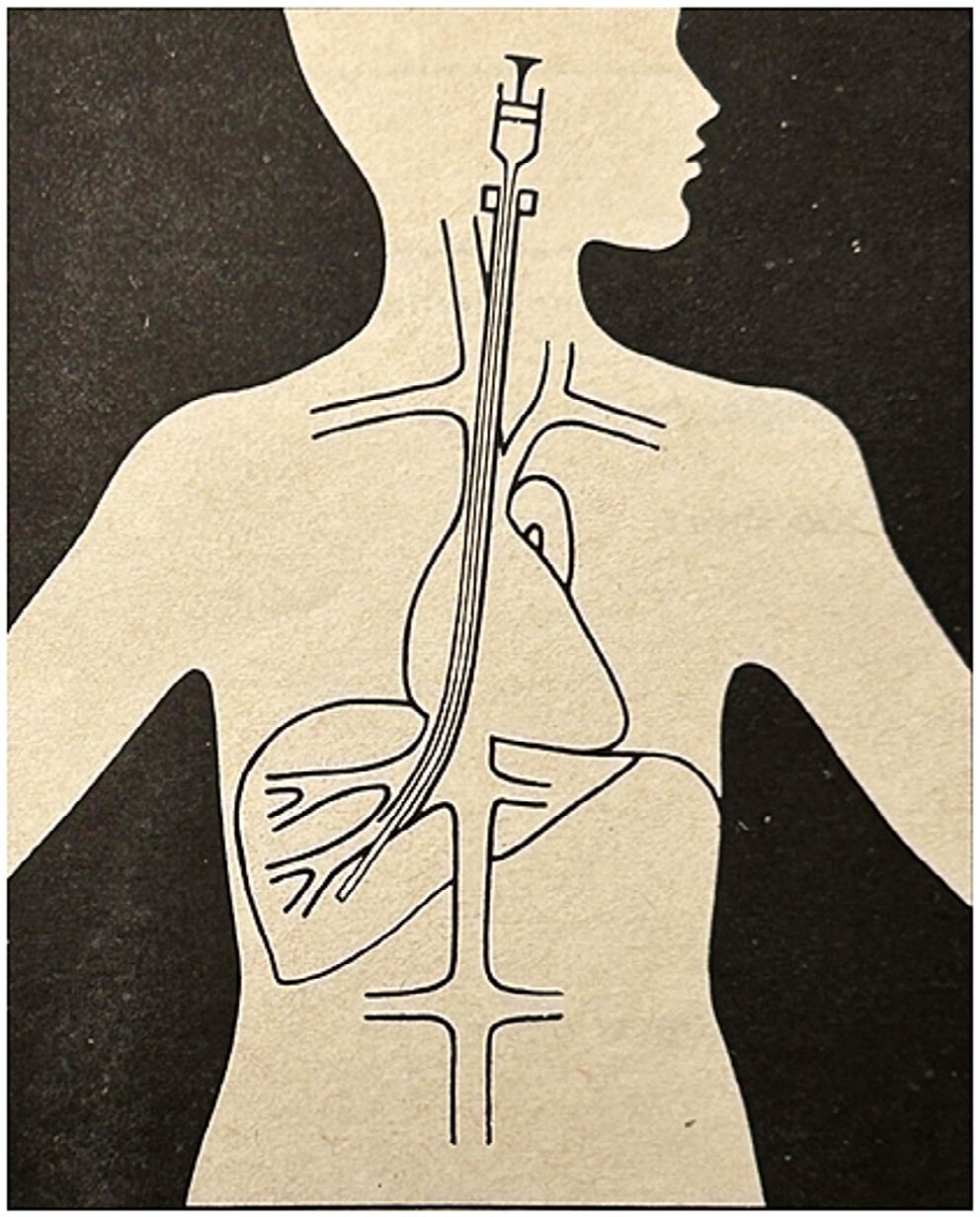
Transjuguloatrial liver biopsy (TJALB) through 9F (or larger) catheter with almost straight course from the jugular venous puncture to the wedged hepatic vein position. Around 1977, from authors' archive.

In 1967, Hanaffe and Weiner performed the first HVC by the transjugular route in order to perform a cholangiography [91]. During this procedure, they observed several obliterations of the needle by liver tissue, and subsequently, they realized the potential of the procedure to obtain liver biopsy. In 1970, they reported the first results on liver biopsies in man performed in connection with the transjugular approach. The following period witnessed five reports on transvenous liver biopsy, see Figure 5, from Universities of Oregon, Paris, London, New York and Copenhagen, comprising 36, 13, 16, 51 and 19 patients, respectively [92, 93, 94, 95, 96]. The delegates at the 12th meeting of the European Association for the Study of the Liver (EASL) in Kavouri, Greece in 1977 were taken by surprise when Didier Lebrec and Jean‐Pierre Benhamou (1927–2008) from Clichy presented transjugular liver biopsies from 300 patients with coagulation defects [97]. Only one fatality was observed, due to capsular perforation during biopsy through an anterior hepatic vein [98]. Five years later, the Clichy group had reached 1000 transjugular liver biopsies [99]. Later, this approach was applied before and after liver transplantation, bone marrow transplantation, and as a routine procedure for liver biopsy. In around 5% of the patients, the common carotid artery was accidentally punctured, but this gave only minor hematoma, and later, with Doppler sonography, it became much easier to identify the jugular vein. Moreover, Lebrec and co‐workers analysed the patients' discomfort during transjugular catheterization and noticed that the procedure was well accepted [100]. In the following years, the transjugular approach to hepatic vein catheterization became a preferred route in several situations and centres. From Copenhagen and Toronto, appeared reports on improved transvenous biopsy needles [101, 102].

After transjugular liver biopsy had become a routine approach in the early 1980s [99] several operators had had the experience of puncturing a portal vein branch with the biopsy needle. The aspiration of very light pink blood, almost arterial, was characteristic of a portal venous position of the tip of the biopsy needle. In 1982, this entailed Ronald Frank Colapinto (1931–2011), Lawrence M. Blendis, and co‐workers from Toronto inserting a Grüntzig^10^ balloon catheter and by inflation to dilate the hepatic tissue and thereby create an intrahepatic porto‐hepatic venous shunt in a patient with cirrhosis and massive haemorrhage [103]. Today, insertion of a Transjugular Intrahepatic Portosystemic Stent Shunt (TIPS) is part of the routine management of patients with severe portal hypertension in most liver centres.

## 5 Flexible Balloon Catheterization of Hepatic Veins

**FIGURE 6:**
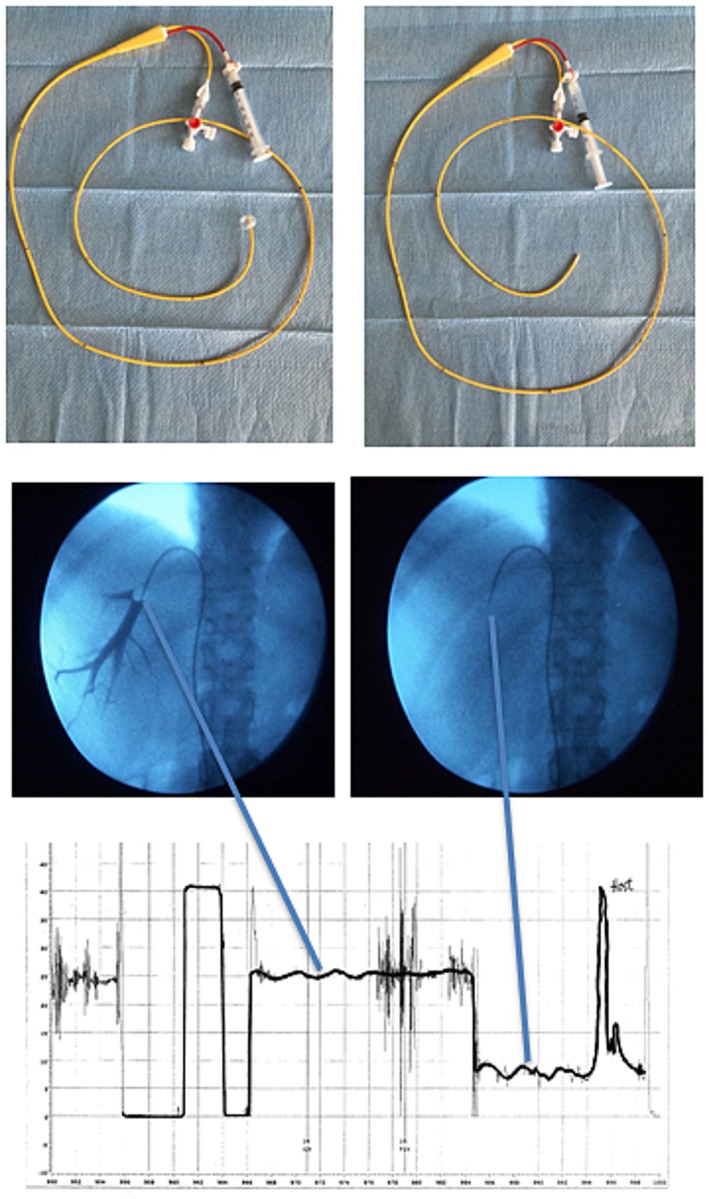
Top: Swan‐Ganz balloon catheter with inflated and uninflated balloon. Middle: Fluoroscopy of hepatic vein catheterization with balloon catheter via femoral route. The inflated balloon occludes the hepatic venous branch which is illustrated by the uncleared radiopack material. Bottom: The pressures in the catheter lumen correspond to the wedged hepatic vein pressure (left) and free hepatic vein pressure (right). The peak to the far right is a cough control. Around 1990, from author's archive.

In 1970, the cardiologists from the Cedars Sinai Center in Los Angles, Harrold James C. Swan (1922–2005) and William Ganz (1919–2009) and co‐workers invented a flexible balloon‐tipped catheter for catheterization of the right heart by flow‐directed passage of the catheter in the main central blood stream [104], see Figure 6. The catheter, now named Swan‐Ganz catheter, was intended to be used without fluoroscopy, only guided by pressure registration, ECG‐monitoring and observation of the catheter length in the body, and thereby ideal at bed‐side and intensive care unit for pressure measurement. The catheter was later on equipped with another lumen, ending 30 cm from the tip, and provided with a thermistor. With a bolus injection of cold saline/isotonic glucose the cardiac output could be determined by thermo‐indicator dilution technique [74, 105]. In addition, they constructed a catheter with a thermistor an inch from the tip for continuous counter‐current infusion of cold saline in order to measure blood flow in the coronary sinus by the Fick‐Stewart principle [106]. It took almost 10 years before these inventions were applied in patients with liver disease.

In 1979, Roberto J. Groszmann, Harold O. Conn (1925–2011) and co‐workers at West Haven, VA Medical Center, Connecticut catheterized 16 dogs and 13 patients with cirrhosis with a Swan‐Ganz balloon catheter and a conventional Cournand catheter [107]. They found a good accordance with wedged hepatic vein pressure and the inflated balloon pressure, and an equally good agreement with the free pressures. In particular the wedged‐to‐free hepatic vein pressure was in excellent agreement to the gradient between the inflated and uninflated pressure with the Swan‐Ganz catheter. They concluded that the balloon technique offered many advantages over the conventional approach, including the ease of achieving and demonstrating the ‘wedged position’ from the femoral route, the ability to measure the free and inflated (wedged) pressure repeatedly without manipulating the catheter, and the measurement of pressure in a larger, more representative segment of the liver. These conclusions turned out to be correct. The balloon catheter technique was soon applied in Barcelona, and it diffused in the next 15 years to most centres throughout the world.

In 1980, the Clichy group presented surprising results on treatment of portal hypertension and bleeding oesophageal varices with the non‐selective beta‐adrenergic blocking agent propranolol [108]. Different types of treatment and prevention were also reported, and soon after the first randomized controlled study [109]. This elicited a cascade of pathophysiological and pharmacological studies all over the world [110, 111, 112].

In 1984, Jaime Bosch (1947–2025) from the Liver Clinic in Barcelona, Spain and Groszmann reported the first catheterization of the azygos vein with measurement of the azygos venous blood flow with the cold water infusion technique, adapted from measurement of the coronary sinus blood flow [113]. Since gastroesophageal collaterals drain into the azygos system, the measurement of blood flow in the azygos vein might provide a quantitative measurement of gastroesophageal collateral blood flow and flow changes in portal hypertensive patients. Five patients with former variceal bleeding had an azygos venous blood flow of mean 600 mL/min, and five patients with surgical portocaval decompression had half this value. Bosch and Groszmann concluded that measurement of the azygos venous blood flow was a rapid, simple^11^ and sensitive method to evaluate blood flow changes in the vessels involved in gastroesophageal bleeding due to portal hypertension. Bosch, Juan Rodés (1938–2017), and co‐workers reported azygos venous blood flow before and after beta‐adrenergic blockade in 23 patients with portal hypertension: mean 544 versus 334 mL/min, a reduction which was greater than the reduction in cardiac output, wedged‐to‐free hepatic vein pressure, and hepatic blood flow [111]. They summarized that the reduction of gastroesophageal collateral blood flow may be the mechanism by which propranolol reduces the risk of repeated episodes of variceal bleeding. Soon the technique was elaborated and undertaken by other centres [114, 115, 116, 117].

In a progress report already in 1978, Dame Sheila Sherlock, Royal Free Hospital, London had concluded on wedged hepatic vein pressure, that ‘This well‐established technique of measuring sinusoidal pressure is now performed less often. It is time‐consuming and is being replaced by the splenic and transhepatic techniques’ [118] (Rarely, had an international expert been more wrong!).

## 6 The Pathogenic Basis of Portal Hypertension and HVC

Although René Laënnec (1781–1826) is best known for the stethoscope, he introduced the term ‘cirrhosis’ for the micronodular condition of a hard and stiff liver abnormality with structural changes of the liver parenchyma, including fibrotic scars. Microscopy of liver tissue had been known for decades from samples obtained during surgery and from autopsies. In the beginning of the 20s century, peritoneoscopy was performed with cystoscopes and tissue samples from the liver could be obtained. However, it was not until Poul Iversen (1889–1966) from Copenhagen in 1943 introduced the percutaneous liver biopsy and later Giorgio Menghini in 1958 invented a better technique that it became routine to obtain specimens from several stages of different liver diseases [77, 119, 120], and the structural changes could now be related to the portal pressure, measured in vivo during HVC, which has played a crucial role in elucidation of the pathophysiology of portal hypertension.

Portal hypertension of the postsinusoidal type was in the 1950s considered the result of a compressed and distorted vascularity, especially with respect to the hepatic venules and veins with a reduced vascular cross‐section area as the outcome [120]. Around 1980, Hector Orrego and Laurence M. Blendis from Toronto reported a correlation of the intrahepatic tissue pressure—which is almost equal to wedged hepatic vein pressure‐ to the amount of collagen in the space of Disse [121]. Later, they reported that hepatocyte enlargement was a major determinant of the increased pressure in cirrhotic and non‐cirrhotic alcoholic liver disease [122]. The mechanism would be that the enlargement of hepatocytes reduced the vascular space and thereby increased the intrahepatic vascular resistance. Investigations by others disclosed no relation between wedged‐to‐free hepatic vein pressure and hepatocyte enlargement in cirrhosis [123]. On the contrary, fibrosis and hepatic architectural destruction were major structural determinants of portal hypertension [124, 125].

In the beginning of the 1990s, several studies showed that vasodilators and vasoconstrictors were operating in cirrhosis [126, 127]. Besides, more static lesions like the cirrhotic nodules, hepatic architectural destruction and scarring, it became evident that the sinusoid endothelial cells and the hepatic stellate cells played an important role in the enhanced hepatic vascular resistance in cirrhosis and probably also smooth muscle cells [128, 129, 130, 131, 132]. Thus, Roberto J. Groszmann and co‐workers performed a large number of experiments with rat models of cirrhosis. The major findings were that especially nitric oxide (NO) modulates hepatic vascular tone in the normal liver as well as in cirrhosis. NO was studied by the presence of the enzyme endothelial nitric oxide synthase (eNOS), which could be isolated from both normal and cirrhotic sinusoidal endothelial cells. It was demonstrated that endothelial‐derived NO modulates portal pressure, and later it was linked to the hyperdynamic systemic circulation in cirrhosis [128, 129, 130, 131]. It was shown that there was too much NO in the systemic circulation and too little NO in the liver where it was needed [131].

Massimo Pinzani and co‐workers from Florence, Italy showed by very elegant combinations of laboratory studies and clinical investigations that the fat storing cells in the liver were pericytes with morphological, ultrastructural and functional characteristics similar to the pericytes, which regulate local blood flow in other organs [132, 133, 134]. They responded to vasoconstrictors like angiotensin II and endothelin‐1 [133]. Hepatic cells such as hepatocytes, hepatic sinusoidal endothelial cells and fat storing cells (hepatic stellate cells) were involved in the hemodynamic changes, especially portal hypertension, impaired degradation of vasoactive substances, production and release of vasodilators and formation of fibrotic tissue. Later it was shown by HVC that the cirrhotic liver actually releases endothelins [135, 136]. By this procedure it was also shown that several fibrotic precursors, vasoconstrictors and vasodilators^12^ were released and degraded in patients with cirrhosis [137, 138, 139].

The success in treating chronic viral hepatitis revealed the remarkable capacity of the liver to modulate scars and to reverse fibrosis, a dynamic capacity which may also be present in different types of cirrhosis. Transient elastography has now been applied for almost 20 years. By this method, it is possible to quantify and serial control the amount of fibrosis in liver disease. This has revealed that the hepatic structure, even in cirrhosis, is much more dynamic and reversible than hitherto considered [140, 141], see later.

## 7 Complications to Liver Disease With Portal Hypertension

In the following sections, distinct complications to advanced liver disease with portal hypertension are considered in more detail, owing to the relevance of catheterization.

### 7.1 Fluid Retention and Ascites Formation in Portal Hypertension

In the 1970s, Laurence M. Blendis from Toronto, Vicente Arroyo from Barcelona and Mauro Bernardi from Bologna were research fellows with Stephen P. Wilkinson and Roger Williams at King's College, London. The investigations were soon directed towards the renin‐angiotensin‐aldosterone system (RAAS) [142]. The main finding was ‘low renin’ cirrhosis. Wilkinson and Williams summarized their findings in a progress report [143], where 2/3 produced ascites with the ‘overflow’ model with expanded extracellular‐ and plasma volume and suppressed RAAS, and 1/3 according to the traditional ‘underfilling’ model with activated RAAS [143]. The first major objection to the ‘overflow theory’ of ascites formation came in 1974 from the effect of a surgical peritoneo‐venous shunt (LeVeen shunt), which both reduced the amount of ascitic fluid and expanded the plasma volume [144].

In 1970, Murray Epstein from Miami, Florida and co‐workers had surprised the medical world by documenting the extreme vasoconstriction present in the kidney in end‐stage cirrhosis, a vasoconstriction which was reversible and became relieved *post mortem* [145]. By isothermic, head‐out water immersion of patients with decompensated cirrhosis, it was possible for Epstein—by the central relocation of the circulating volume—to suppress the RAAS in almost all patients, although only half of the patients showed a natriuretic response [146]. Thus, other salt‐water‐retaining mechanisms would also operate.

In the years 1979–1981, Arroyo, Bosch, Rodès and co‐workers from Barcelona reported important results on the RAAS, measured in patients with cirrhosis during HVC [147, 148]. They showed that the circulating renin and aldosterone ranged from normal to extremely high values in patients with ascites and hepatorenal syndrome, values that were not due to reduced hepatic degradation, and that high plasma renin was a prognostic indicator in non‐azotemic cirrhotic patients with ascites [148].

Establishment of circulating noradrenaline (NA) as an index of overall sympathetic nervous activity (SNA) [149] prompted Jens H. Henriksen and Helmer Ring‐Larsen from Copenhagen to investigate catecholamines in patients with cirrhosis. During a HVC, samples were obtained from different organ veins and systemic artery. The results showed circulating NA from normal to exceptionally high values in patients with cirrhosis, highest in the renal vein, indicating a preferential renal sympathetic overactivity in patients with advanced disease [150]. Subsequent, relations to disease state, organ extraction, hemodynamics, prognosis and renal blood flow were investigated [151, 152, 153, 154]. Robert W. Schrier (1936–2021) and co‐workers from University of Denver, Colorado soon confirmed the results and found a relation to the impaired sodium and water excretion in these patients [155], and Gerald F. DiBona from University of Iowa reported increased renal neural activity in hepatorenal syndrome [156]. All indicating a major role of enhanced sympathetic nervous activity in salt‐water retention in advanced cirrhosis.

In 1981, an atrial natriuretic factor/peptide (ANP) was identified from the cardiac auricle and Alexander L. Gerbes from Munich determined the circulating values of ANP in patients with cirrhosis [157], but the role of ANP here was not clear [158], neither in supine patients nor after central loading [159], where the natriuretic response was dissociated from the increase in ANP. The ANP in cirrhosis turned out to be very complex.

#### 7.1.1 Peripheral Arterial Vasodilatation Hypothesis in Portal Hypertension

By injecting ^125^I‐labelled human serum albumin into the right atrium during a HVC, and subsequent serial blood sampling from the aortic bifurcation, it was possible to determine the central and arterial circulation time,^13^ the cardiac output, and the blood volume in the heart, lungs and central arterial tree up to points temporally equidistant to the aortic bifurcation. In 1986, Henriksen, Flemming Bendtsen and co‐workers reported a contracted central and arterial blood volume in supine patients with cirrhosis (1.45 L vs. 1.83 L in controls), determined by this advanced technique [161].

In 1987, Arroyo, Bernardi, Epstein, Henriksen, Rodès and Schrier met in Barcelona to discuss fluid retention and kidney function in cirrhosis with portal hypertension. The proposed ‘peripheral arterial vasodilatation hypothesis’ was based on the presence of a hyperdynamic systemic circulation with increased cardiac output, increased splanchnic inflow, reduced splanchnic and peripheral vascular resistance, and counter‐regulatory activation of the sympathetic nervous activity, overactivity in the RAAS and increased non‐osmotic release of vasopressin in advanced cirrhosis [162, 163]. A peripheral arteriolar vasodilatation would result in reduced arterial blood pressure and increased blood flow in several vascular beds, except the kidneys [164], which would reduce the arterial compliance.^14^

Henriksen, Bendtsen and Møller elaborated the HVC‐kinetic method to determine the central and arterial blood volume and arterial compliance [126, 165, 166, 167, 168, 169, 170]. These HVC‐investigations during more than 10 years^15^ gave major experimental evidence for the peripheral arterial vasodilatation hypothesis. In addition, the results suggested the presence of an incomplete cardiac function in patients with advanced cirrhosis (see subsequent section).

### 7.2 Cirrhotic Cardiomyopathy

As earlier mentioned, the first report on hyperdynamic circulation in cirrhosis appeared in 1953 [73]. Apart from the increased heart rate and low arterial blood pressure, these patients showed no signs of overt cardiac failure; however, clinicians have often considered the heart and the liver related. In the 1980s, it became clear that a severely reduced survival was seen in cirrhotic patients with highly elevated circulating catecholamines [154]. Cohn and co‐workers [171] and Kelbæk and co‐workers [172] found reduced cardiac performance during physical exercise in patients with cirrhosis, findings confirmed by others [173]. In 1994, Møller and Henriksen reported enlarged left atrium, marginally enlarged left ventricle, and contracted right‐sided chambers in the heart of patients with cirrhosis [170], and the following year, it was shown that the systolic function was reduced during an osmotic challenge in patients with advanced disease [169]. In 1994, Samuel S. Lee and co‐workers from Calgary, Alberta, Canada, reported changed beta‐adrenoreceptor function in rats with experimental cirrhosis [174] and in 1996, they reported altered beta‐receptor signal transduction in the pathogenesis of cardiac dysfunction. In the same year, they introduced the term ‘cirrhotic cardiomyopathy’ [175, 176].

*Cirrhotic cardiomyopathy* denotes a chronic cardiac dysfunction in cirrhosis, characterized by blunted contractual responsiveness to stress, altered diastolic relaxation and electro‐mechanical abnormalities such as prolongation of the QT‐interval [177]. Major contributions to the understanding of cirrhotic cardiomyopathy have been gained through hepatic vein pressure‐volume measurements, collection of bioactive substances and biomarkers, elucidation of electro‐mechanic relations, and analysis of the coupling between the left ventricle and the arterial tree [17, 178, 179, 180, 181, 182].

**FIGURE 7:**
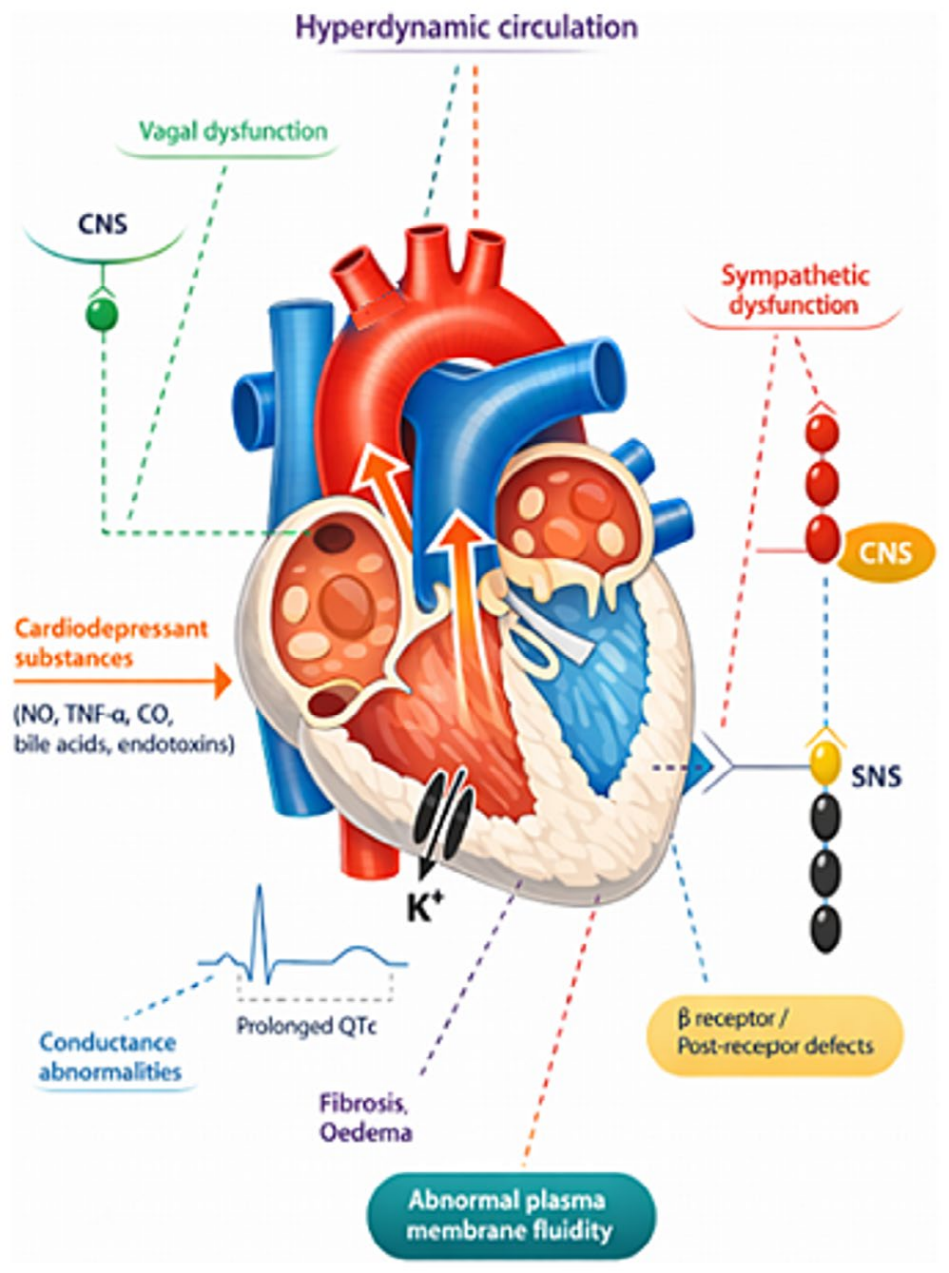
Cirrhotic cardiomyopathy illustrated with autonomic dysfunction and characteristic changes in diastolic and systolic function and membrane, receptor, myocardial and conductive components. Redrawn from Møller and Henriksen 2002 [181] and 2008 [183], authors' archive.

Several characteristics of systolic‐, diastolic‐ and neuro‐cardiac dysfunction are present, see Figure 7. Especially the long QT‐syndrome has attracted much attention, in part owing to the challenges of frequency correction [180, 184, 185, 186]. The increased circulating levels of the cardiac peptides, proBNP and BNP, were investigated during HVC [184, 187].

During late systole the pulse reflection wave in the large arteries and aorta is damped in advanced cirrhosis, and this has been analysed by combined hepatic vein‐ and arterial catheterization and shown to have a frequency and amplitude pattern which may protect the heart [182], and may thereby explain why patients with cirrhotic cardiomyopathy often do not have symptoms of heart failure during rest, but become overt during stress, like exercise, orthostatic manoeuvres and pharmacological manipulation.

Cirrhotic cardiomyopathy is still under investigation [183, 188, 189, 190, 191, 192], factors acting at the molecular and cellular level include inflammation, membrane lipids, receptors and signal transduction, NO, CO, cytokines and isoform change of myosin, and new diagnostic modalities and advanced imaging analysis have disclosed interesting patterns [190, 191, 192, 193]. Cirrhotic cardiomyopathy, especially the diastolic dysfunction, may affect the prognosis and aggravate the course of the disease during stressful and invasive procedures such as insertion of TIPS and liver transplantation [190, 194]. Finally, it seems that decreased cardiac function and autonomic dysfunction are major issues in the development of irreversible renal failure in patients with advanced cirrhosis, and a *cardiorenal syndrome* has been put forward [195, 196]. For further information, see reviews [189, 196].

## 8 Hepatic Vein Catheterization Today

HVC with measurement of the hepatic venous pressure gradient (HVPG) is now the gold standard in posthepatic‐ and postsinusoidal portal hypertension. At the Baveno V and VI meetings in 2010 and 2015, relatively few concrete guidelines were given with respect to the measurement of HVPG [197]. By contrast, in 2021 at the Baveno VII meeting, the relevance and indications for measuring the HVPG as a gold standard were discussed intensively, and new and updated recommendations were given [2]. Detailed guidelines were offered for HVPG measurements with respect to practical performance, type of catheter, speed of pressure tracing, calibration, spatial placement of the catheter tip within the hepatic vasculature, and limits for pressure intervals with respect to classification of portal hypertension, prognostic and therapeutic consequences [2]. This also held true for medical treatment of portal hypertension and treatment with TIPS. These guidelines are summarized in Table 2. It is beyond the scope of this review to go into details, but readers should consult recent expert reviews [198, 199].

**TABLE 2:** Recommendations for hepatic vein catheterization and pressure measurements from the Baveno VII Meeting in 2021.

During hepatic vein catheterization, the best measurements are obtained by an end‐hole, compliant balloon occlusion catheter. A small volume of contrast medium should confirm the satisfactory occluded position of the catheter.
Pressure should be measured with calibrated and linear transducers and a slow speed, permanent tracer recordings. The wedged pressure requires a stabilization time of minimum 1 min. All pressures should be recorded in triplicate.
The wedged‐to‐free hepatic vein pressure gradient (hepatic venous pressure gradient) has superior clinical prognostic value compared to a reference point in the right atrium or the inferior vena cava which might be used alternatively.
The free hepatic vein pressure should be measured within 2–3 cm of its confluence with the inferior vena cava. The pressure in the inferior vena cava may be measured as an internal control.

*Source:* From [2, 197, 198, 199].

Since the landmark paper by Gluud and co‐workers in 1988, it has been known that the HVPG contains important prognostic information with respect to survival [200]. Now at the Baveno VII this has been extended to prevention of first bleeding and re‐bleeding from oesophageal varices, development of ascites, other types of decompensation, and prediction of development of hepatocellular carcinoma, independently of the severity of liver disease [2, 201]. Accordingly, the whole symposium was labelled ‘Personalized Care for Portal Hypertension’.

With the recognition of dynamic fibrosis in liver disease [202] a major interest has been turned towards quantification of fibrosis by various non‐invasive measurements of reflections and imaging patterns. This has led to major new insight into the dynamic process of structure changes in liver disease [203, 204]. These patterns may also be correlated to the size of HVPG/portal pressure and with AI, information on the size of portal pressure may become obtained from special algorithms. However, it is beyond the scope of this survey to go into details with these fascinating new issues.

## 9 Discussion

HVC has for 80 years been an important tool in the scientific investigation of liver disease, portal hypertension and its numerous complications. Several comments and discussions have already been offered in the foregoing sections. Investigators like Stanley E. Bradley, Dame Sheila Sherlock, Roberto J. Groszmann, Jaime Bosch, Didier Lebrec and Guadalupe Garcia‐Tsao should be emphasized for their life‐long, strong and renewing engagement in HVC and portal hypertension.

HVC followed the right‐sided heart catheterization by different time lags.^16^ With the local splanchnic kinetics, Bradley was in advance of other cardiovascular kinetics [26, 87]. Resistance against heart catheterization and HVC in lay public and among professionals has been documented in the USA, France and UK [4, 6, 15, 16]. It included several worries: invasive and sterile procedures, potential complications like infection, bleeding, arrhythmia, vasovagal syncope, expensive equipment, time‐consuming procedures, difficult pressure measurements, doubtful clinical significance, etc.^17^ A lot was intuitively understandable, but not in proportion.

Results, utilization and potentials of HVC are summarized in Table 3.

**TABLE 3:** Diagnostics, information from, results, utilization, use and clinical application of catheterization of hepatic veins and other vascular compartments.

Flow measurement (indirect Fick, thermodilution, indicator dilution)
Pressure measurement (wedged catheter, free catheter, balloon occluded catheter)
Angiography
Liver biopsy (TJALB)
Elective blood samples (substance identification, extraction, release)
Therapy (medical, TIPS, surgical)
Therapy control (portal hypertension, TIPS, paracentesis)
Pathophysiology (portal hypertension, collateral circulation, cardiomyopathy, lung disease, systemic circulation, vasodilatation, volume distribution, compliance, vascular permeability, etc.)

### 9.1 Pressure Measurement

In the early and mid‐1940s, dynamic pressure measurement was very difficult with the enormous Hamilton manometer [16]. In the late 1940s, Erik Warburg (1892–1969) and Anders Tybjærg Hansen (1915–2006) invented the capacitance (condenser) manometer for dynamic cardiovascular pressure measurement [18]. Later came strain gauge manometers and ink‐jet registration, and from around 1960 all pressure measurements were fast and reliable from a technical point of view. Although wedged‐ and free hepatic venous pressures in theory might be measured by a static water column, it is important to apply full dynamic registration, due to detection of artefacts, characteristic patterns and repeated measurements, as specified in the recommendations [2, 198]. To days widespread pressure measurements in intensive care units, anaesthetic units, operation rooms, coronary and physiologic labs, interventionary radiology rooms, gastro labs, etc. have expanded the possibilities for HVC if the medical expertise and routine are present. Despite this, there is still today some resistance against the procedure, which may be considered mainly investigative and not always necessary [197], if the patient for example has had an esophagoscopy that has revealed the presence of oesophageal varices and thereby most likely a HVPG above 12 mmHg.

### 9.2 Blood Samples

Numerous important scientific results have been obtained by selective blood samples from hepatic and other organ veins (kidney, adrenal glands, brain, coronary sinus, azygos vein) [19, 29, 89, 137, 150, 155, 161]. The blood samples have been analysed for metabolites, hormones, other bioactive substances, test substances, tracers, indicators and dyes.^18^

### 9.3 Diagnostics and Therapy

Routine HVCs were performed in increasing numbers in several centres from mid‐late 1950s. The drop in surgical poto‐systemic operations in the 1970s together with the beginning of large‐scale prognostic‐ and therapeutic trials of other treatments (e.g., sclerotherapy) changed the indications for HVC. Imaging diagnostics also influenced the need of HVC. New standards and overwhelming documentation for precision, accuracy, and diagnostic‐ and prognostic security have now turned the HVPG into the gold standard for assessing portal hypertension [2].

Today the indications for routine HVC are as follows: (1) Diagnostic classification of portal hypertension (prehepatic/sinusoidal, intrahepatic, postsinusoidal/hepatic); (2) differential diagnostics (e.g., heart failure^19^); (3) determination of the degree of portal hypertension (choice of treatment); (4) Prognostic evaluation (risk of bleeding, rebleeding, ascites formation, encephalopathy, mortality); (5) Monitoring of treatment (e.g., non‐selective beta‐adrenergic blocking agents); (6) Prior to invasive treatment (like TIPS, surgery, transplantation); and (7) the Control of such procedures. HVC with pressure measurement is mandatory before and after surgical porto‐systemic shunts. The evaluation of type and magnitude of portal hypertension is important before and sometimes during medical therapy. The separation of responders versus non‐responders assumes a very precise pressure measurement and thereby determination of the HVPG.

TIPS is often preceded by an ordinary HVC and controlled by Doppler‐sonography and HVC [205]. The placing of the stent by transjugular hepatic vein catheterization allows pressure measurements [206], although the patient may be under general anaesthesia and thereby have somewhat lower pressure levels. In Budd‐Chiari syndrome, a HVC with the missing placement of the catheter tip in hepatic veins may lead to the diagnosis and thereby the right therapy [207]. It should also be kept in mind that portal hypertension may ameliorate along with a spontaneous improvement of liver function, an amelioration which can be detected, owing to the high precision of HVPG determination [2, 208].

Non‐invasive diagnostics like transient elastography, biomarkers and diagnostic imaging modalities [202, 209] may aid screening and control, but cannot replace HVC and HVPG measurements [2, 198, 199].

## 10 Conclusion

HVC in man was first performed 80 years ago. Five years passed before wedged pressure measurements were performed and routine HVCs were done in liver patients. Despite substantial developments in route, catheter type, pressure measurements and registration, fluoroscopy, transvascular technique, etc., the basic concepts are the same today. New advanced technology, materials, imaging techniques and IT power have, together with the inborn dynamics of the catheterization, given a progressive development, and there are potentials of further refinement and applications in science, clinical physiology, gastroenterology and hepatology.

## Author Contributions

Idea and first draft (J.H.H.); all authors have contributed to all parts of the manuscript.

## Disclosure

No use of AI has been applied in the preparation of the manuscript.

## Conflicts of Interest

The authors declare no conflicts of interest.

## Data Availability

The data that support the findings of this study are available from the corresponding author upon reasonable request.

## Endnotes

^1^
Ballistocardiography was a method for cardiac output determination which applied registration of the distribution of weight and recoil in the body.

^2^
It is relevant to notice that the drive to these investigations was to obtain blood samples from different parts of the circulation, not primarily to determine the vascular pressure. Dynamic pressure measurements were in the beginning of the 1940s a difficult and time‐consuming procedure. William Hamilton (1893–1964) had in 1934 constructed a dynamic manometer, a reliable but large instrument of the size of 1–2 m^3^.

^3^
The roentgenographically demonstrated ‘stricture’ in the upper part of the inferior vena cava in patients with ascites was in part functional, owing to a Venturi‐effect of the high linear velocity of blood at the cavo‐atrial junction in the ascitic state and disappeared after paracentesis [46].

^4^
Later Bradley's kinetics has been recognized as one of four basic kinetic systems for determination of mean transit time and mean sojourn time in bodily systems. His kinetics is termed: ‘The inlet–outlet tracer infusion principle’, which now has been described in several textbooks on tracer kinetics [47, 48].

^5^
It is present in normal man, but especially pronounced in patients with portal hypertension [49, 50]. The Bradley principle has also been applied by Liberman and co‐workers to determine the lymphatic mean sojourn time, and thereby the volume in the lymph system from intestines and liver to the inlet of the thoracic duct in the venous system [51]. The method has been applied to determine the increased dispersion of blood flow in parts of the vascular system.

^6^
Later it was recognized that bromsulphalein may give serious allergic reactions, for which reason it is not used today.

^7^
In 1948, William Hamilton's group and André Cournand's group compared the direct Fick‐measurement of cardiac output to that of the indicator dilution technique by Evans Blue in man [74]. They found an excellent agreement between the two methods, and from then on the dye dilution technique was considered to be a standard method to determine cardiac output in man.

^8^
The three ‘columns’ of microvascular perfusion were (1) Starling's filtration theory, (2) Krogh's recruitment phenomenon and (3) the pore‐theory of Pappenheimer. The ‘columns’ explained the transport of blood along the capillary tube as well as the transcapillary exchange of matter. Already, Starling suggested a hierarchy of capillaries with those in the liver being the most permeable. This was studied in detail by Gunnar Grotte, who used dextrans of different molecular sizes to quantify the functional structure of the liver capillary wall. In 1959, Bennett, Luft and Hampton confirmed the open structure of the normal liver sinusoids without a basement membrane by their electronic microscopy investigations. Later, Kardon and Kessel contributed much to the understanding of the three‐dimensional organization of the normal hepatic microcirculation by their cast studies.

^9^
Kenneth L. Zierler (1917–2009), professor of Medicine and Physiology at John Hopkins University, Baltimore had earlier invented and analysed the fundamental relations between blood flow, blood volume and mean circulation time, when injecting a tracer into the arterial organ inlet and collecting samples from the venous outlet from the organ [87, 88].

^10^
Andreas Grüntzig (1939–1985) was a successful pioneer in Zürich (1971–1985) in catheter‐construction and transvascular angioplastic on coronary arteries.

^11^
However, most would agree that catheterization of the azygos vein demands some skill.

^12^
The potential circulating vasodilators were Nitric oxide, Calcitonin gene‐related peptide, Atrial natriuretic peptide, Brain natriuretic peptide, Natriuretic peptide type C, Endocanabinoids, Endothelin‐3, Interleukins, Tumour necrosis factor‐alpha, Thromboxane‐A2, Leukotriens, Prostacyclin, Adrenomedullin, Adenosine, Carbon monoxide, Hydrogen sulphide, Bradykinin, Enkephalins, Glucagon, Histamine, Substance P, Vasoactive intestinal polypeptide.

^13^
The concept of mean circulation (transit) time was elaborated by Francis P. Chinard (1918–2015) and co‐workers to the central vascular compartments [160], and later adapted by Goreskey, Huet and others.

^14^
Arterial compliance is increase in arterial volume relative to increase in pulse pressure.

^15^
The reduced central and arterial blood volume (CBV) turned out to be inversely related to the hepatic venous pressure gradient, the peripheral vascular resistance, the mean arterial blood pressure, and the severity of disease, as evaluated by the Child classes A, B, C and to the circulating level of the potent vasodilator calcitonin gene‐related peptide. Hydrostatic manipulation of patients with cirrhosis and control subjects changed CBV as expected. CBV could be manipulated by oxygen inhalation, beta‐adrenergic blockade, the vasoconstrictor terlipressin, the somatostatin analogue octreotide and by hyperosmotic‐ and albumin infusions. By albumin expansion it was shown that Child class A patients reacted almost as controls, but those with advanced cirrhosis (class C patients) could not change their arterial compliance. By osmotic volume expansion, it was earlier demonstrated that in control subjects and in patients with cirrhosis, class A, the volume expansion was evenly distributed throughout the circulation. However, in class C patients it was not possible to expand the central and arterial blood volume significantly. As the central vascular transit time remained low (analogue to Cohn and Groszmanns invetigations in the beginning of 1970s) the results pointed to a cardiac output, although increased, but not sufficiently so to satisfy the demands of the body.

^16^
Blood samples: 4 years, Pressure measurements: 7 years, Balloon catheter application: 9 years, Puncture: 11 years, Cold water blood flow (thermodilution technique): 13 years, Systemic transit kinetics: 14 years, Catheter therapy: 18 years.

^17^
Radiologists did not like pressure measurements and cardiologists did not like liver patients, especially not in the intensive care unit. The heart was a noble organ and should be snake/hose/catheter impervious. The liver was a type of cesspool and not worth a catheter.

^18^
Bromsulphalein, indocyanine green, ethanol, galactose, glucose, lactate, insulin, C‐peptide, glucagone, adrenaline, noradrenaline, renin, leptin, ANP, BNP, proBNP, CNP, endothelin‐1, endothelin‐3, HIDA, etc.

^19^
When cardiac edema and ascites do not respond as expected on a given therapy, it is sometimes because the genesis is hepatic (and vice versa). HVC can solve that (this also holds true when the ascites turns out to be malignant).
